# Feasibility of a Person-Centred Nursing Model Targeting Patient and Family Caregiver Needs in Allogeneic Haematopoietic Stem Cell Transplantation

**DOI:** 10.3390/healthcare13192463

**Published:** 2025-09-28

**Authors:** Annika Malmborg Kisch, Linda V. Eriksson, Anna O’Sullivan, Aida Shahriari, Karin Bergkvist, Carina Lundh Hagelin, Jeanette Winterling

**Affiliations:** 1 Department of Health Sciences, Lund University, 221 00 Lund, Sweden; 2 Department of Haematology, Oncology and Radiation Physics, Skåne University Hospital, 221 85 Lund, Sweden; 3 Department of Neurobiology, Care Sciences and Society, Division of Nursing, Karolinska Institutet, 171 77 Stockholm, Sweden; linda.eriksson@ki.se (L.V.E.); carina.lundh-hagelin@mchs.se (C.L.H.); jeanette.winterling@regionstockholm.se (J.W.); 4 Department of Health Care Sciences, Marie Cederschiöld University, 116 28 Stockholm, Sweden; anna.osullivan@mchs.se; 5 Department of Nursing Sciences, Sophiahemmet University, 114 86 Stockholm, Sweden; 6 Karolinska Comprehensive Cancer Centre, Medical Unit HHLH, Karolinska University Hospital, 171 77 Stockholm, Sweden; aida.shahriari@regionstockholm.se; 7 Department of Public Health and Caring Sciences, Uppsala University, 751 23 Uppsala, Sweden; karin.bergkvist@uu.se

**Keywords:** allogenic haematopoietic stem cell transplantation, cancer, family caregivers, feasibility, patients, person-centred nursing

## Abstract

**Background/Objectives**: Undergoing intensive cancer treatment, such as allogeneic haematopoietic stem cell transplantation, challenges the entire life situation of patients and their family caregivers. A prerequisite for effective care is that interventions are tailored to the unique circumstances of each individual. The aim of this study was to examine the feasibility of a developed person-centred nursing model in this context. **Methods**: The nursing model involves systematic use of conversation tools with subsequent conversations to assess and address the needs of patients (two tools) and their family caregivers (one tool). Patients, family caregivers and registered nurses from two Swedish stem cell transplantation centres testing the model were included. Data to measure practicality were gathered from the tools and documented conversations, while acceptability was assessed from the interviews. **Results**: 36 patients, 32 family caregivers and 16 registered nurses participated. 67–94% of the patients and 94% of the family caregivers completed the tools. 78–97% of the subsequent conversations were conducted as planned. 78% of the patients and registered nurses were positive about one of the patient tools, while 41% were positive about the other patient tool. 95% of the family caregivers and registered nurses were positive about the family caregiver tool. Most participants considered that the systematic use of tools helped to structure a holistic needs assessment. **Conclusions**: This feasibility study indicates that most components of our nursing model are practical, acceptable and support registered nurses when conducting person-centred nursing. With further development, the model has the potential to enhance the quality of care and support within the cancer context.

## 1. Introduction

Being diagnosed with cancer and undergoing intensive treatments such as allogeneic haematopoietic stem cell transplantation (allo-HCT) challenges a person’s entire life situation, as well as the situation of their family caregivers [1,2,3,4,5,6]. However, all individuals are unique, and each person reacts to and copes with the situation differently. Allo-HCT is an intensive curative treatment primarily for haematological malignancies with a significant risk of relapse and severe complications, including graft-versus-host disease and complex infections [7,8]. The treatment trajectory begins with an initial hospitalisation period of 4–6 weeks, followed by an intensive post-discharge care phase lasting approximately three months and continuing with a prolonged rehabilitation period of 6–12 months. Throughout the allo-HCT process, patients face multifaceted challenges that give rise to physical, psychological, existential and social needs [3,7,8,9]. These needs are highly individual and dynamic, varying in type and intensity both between patients and over the course of the treatment trajectory [10].

The social network of family and friends, as well as the healthcare team, have been shown to be vital in the care of allo-HCT patients [11]. At the same time, the life situation of family caregivers is profoundly impacted as they navigate their own worries about living with a seriously ill relative [11], as well as assuming significant responsibilities, providing both physical and psychological support to the patient [12]. The needs of family caregivers are also highly individual and evolve over time [5].

To provide patients with the most effective care, medical and nursing interventions must be carefully tailored to the unique circumstances of each individual. This highlights the importance of person-centredness as a key approach to meeting the complex and evolving needs of both patients and their family caregivers [13,14]. By integrating the patient’s perspective, experiences and individual needs into the care process, patient participation, trust and a more holistic recovery can be promoted. Registered nurses (RNs) play a pivotal role in this process, as they are uniquely positioned to assess and manage patient symptoms and offer person-centred support to both patients and family caregivers [15,16]. Although the importance of person-centred care is widely recognised, to our knowledge no studies have systematically evaluated structured nursing models that integrate both patients and family caregivers in the context of allo-HCT. However, other supportive interventions aimed at enhancing the well-being of patients and their family caregivers have been explored. For example, Pralong et al. (2023) developed a supportive palliative care intervention that takes account of patients’ and caregivers’ experiences and needs—particularly existential concerns, to improve symptom management, communication and quality of life during and after treatment [17].

**Figure 1:**
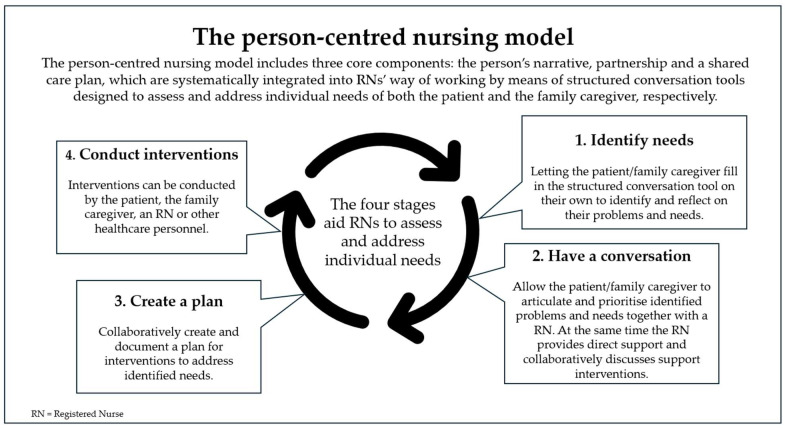
The person-centred nursing model.

In this research project, we developed a person-centred nursing model designed to address the complex and multifaceted individual needs of both patients and family caregivers within the clinical context of allo-HCT, ensuring a relevant and holistic approach to care. Its development is grounded in established theories, previous research, clinical and pedagogical expertise, as well as end-user perspectives. The process was guided by the Medical Research Council (MRC) framework for the development and evaluation of complex interventions [18], with particular focus on ensuring applicability in clinical settings. Therefore, the model has been developed using Experience-based Co-Design (EBCD), a participatory research approach that brings together healthcare staff and patients to collaboratively improve the quality of care [19]. Key stakeholders in the research project include managers, “Champions” (selected RNs who have a central role in the project by functioning as a link between the clinics and researchers), patients and family caregivers at the two largest allo-HCT centres in Sweden participating in the research project. Our person-centred nursing model is inspired by McCormack and McCance’s person-centred nursing theory [20] but based on the three core components of the University of Gothenburg Centre for Person-centred Care (GPCC): the patient’s narrative, partnership and a shared care plan (GPCC) [21]. These core components are systematically integrated by using structured conversation tools designed to aid RNs to assess and address individual needs of both the patient and family caregivers. The use of these conversation tools involves four stages: (1) introducing the tool to the patient and family caregiver separately to encourage them to reflect on their current problems and needs, (2) engaging patients and family caregivers in structured conversations with an RN, where they can articulate and prioritise their own identified needs while the RN facilitates the conversation in a collaborative dialogue, recognising the expertise of the patient or family caregiver as an essential component of the partnership, (3) collaboratively creating a shared care plan that outlines specific support interventions, which may be addressed immediately, managed independently by the patient or family caregiver, or coordinated with the healthcare team, and (4) conducting nurse-led support interventions to address the identified needs, including preparing and supporting patients and family caregivers to manage side effects, needs and distress (Figure 1).

**Figure 2:**
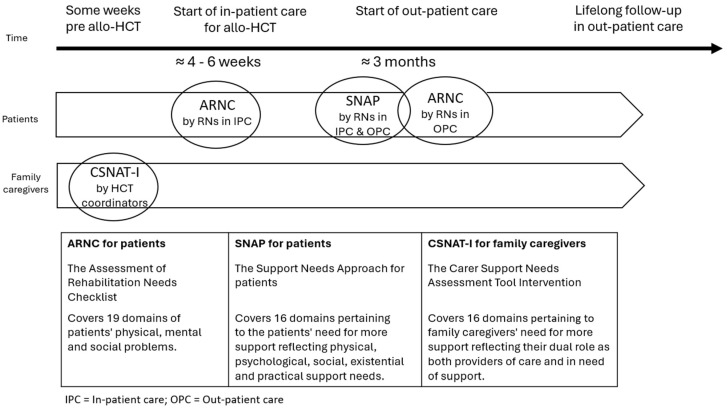
Description and time points for the three conversation tools included in the person-centred nursing model.

To capture individual problems and needs of both patients and family caregivers three conversation tools were selected to be used at different time points in our person-centred nursing model (Figure 2). To the best of our knowledge, no previous studies have incorporated different previously tested conversation tools in one nursing model. Two conversation tools—The Assessment of Rehabilitation Needs Checklist (ARNC) [22] and The Support Needs Approach for patients (SNAP) [23]— were selected for patients, while one conversation tool, The Carer Support Needs Assessment Tool Intervention (CSNAT-I), was selected for family caregivers [24,25]. The ARNC, developed in Sweden, encompasses 19 domains of patients’ physical, mental, social and existential problems. Recommended by the National Care programme for Cancer Rehabilitation in Sweden as a tool to assess cancer rehabilitation needs in patients, it is currently being implemented in Swedish cancer care (RCC 2020) and has been validated in a Swedish cancer population [22]. The SNAP, developed in the UK for patients diagnosed with chronic obstructive pulmonary disease [23], includes 16 domains covering the patients’ self-identified physical, psychological, social, existential and practical support needs, and was recently translated into Swedish and validated [26]. The CSNAT-I, also developed in the UK in the context of palliative care, includes 16 domains pertaining to family caregivers’ self-reported support needs, reflecting their dual role as both providers of care and persons in need of support [24,25]. It has been translated into Swedish and validated in a Swedish context [27] and applied in previous studies by our research group on support for family caregivers in allo-HCT [1,5,28]. A brochure about available support for family caregivers was developed and tested in our previous feasibility study of the CSNAT-I [28] and is now available at the two Swedish allo-HCT centres where this study was conducted. Our nursing model also includes a short web-based training for RNs about person-centred nursing, how to use the three conversation tools, conduct the subsequent conversations and create a shared care plan. RNs receive the training before starting to use the tools.

The aim of this study was to examine the feasibility of a person-centred nursing model targeting patient and family caregiver needs in the allogeneic haematopoietic stem cell transplantation context.

## 2. Materials and Methods

### 2.1. Design

We conducted a single-arm, mixed-methods feasibility study over six months (March–September 2023), following the Consolidated Standards of Reporting Trials (CONSORT) 2010 extension checklist for pilot and feasibility trials. The study focused on two key areas of feasibility described by Bowen et al. (2009): practicality, i.e., the extent to which the model could be applied as intended, and acceptability, i.e., the extent to which participants using the model find it appropriate and satisfactory [29]. Practicality was examined by measuring how many participants completed a tool and how many had a follow-up conversation with an RN [29]. Acceptability was examined by analysing the extent to which patients, family caregivers and RNs who used the model found the tools and conversations appropriate and satisfactory.

### 2.2. Sample and Procedure

Participants were consecutively recruited from March to September 2023 from the two largest HCT centres in Sweden, in Stockholm and Lund. Inclusion criteria were adult (≥18 years) patients planned to undergo allo-HCT and one of their adult (≥18 years) family caregivers who were able to read and speak Swedish and without cognitive impairment. Exclusion criteria were minors (<18 years), individuals unable to read and speak Swedish and individuals with cognitive impairment. Once a patient became eligible for transplantation and a plan for the allo-HCT had been established, patients who met the inclusion criteria were briefly informed about the study. This process took place continuously. The HCT coordinators, all of whom were RNs, informed the patients and asked them to nominate a family caregiver involved in their everyday life. With the patient’s consent, the HCT coordinator forwarded the contact details of the patient and family caregiver to the study coordinator, who then contacted the patient by telephone to provide more detailed information about the study and invite them to participate. If the patient agreed to participate, they were asked to permit the study coordinator to contact the family caregiver to invite them to participate as well. Upon agreement, written study information was provided by post. If the patient consented, the family caregiver received both written and oral information about the study and was invited to participate.

### 2.3. Data Collection

Data from using tools and conducting subsequent conversations was collected by gathering all completed conversation tools, as well as data from the RNs’ documentation in the patients’ medical records from conversations conducted using the ARNC [22] and SNAP [23,26]. Additionally, data from the RNs’ documentation when using the CSNAT-I [24,25,27] were collected. No modifications were made to the predefined criteria or procedures for assessing the practicality or acceptability of the model after the start of the study.

Semi-structured individual telephone interviews were conducted with patients and family caregivers by three of the authors (AO, CL, KB) and one Ph.D. student using an interview guide. The interviews were intended to evaluate the participants’ experiences of the model and its different parts and whether they found it appropriate and satisfactory. Patients and their family caregivers were consecutively asked to participate in interviews until approximately 15 patients and 15 family caregivers and a fairly equal number of participants of both sexes were included. Patients were also asked about experiences of self-care, reported in another paper.

Semi-structured focus group interviews, conducted either digitally or in person, were held with RNs who had used the conversation tools in patient or family caregiver conversations. Convenience sampling was used for the inclusion of RNs, i.e., those who were available and willing to participate. The interviews were led by three of the authors (AK, AO, LE) and one PhD student, using an interview guide. The interviews were intended to evaluate the RNs’ experiences of the model and the different parts of the model. The interview guides for patients, family caregivers and RNs were developed with the aim of gathering their experiences of using the conversation tools and conducting the conversations. Patients and family caregivers were asked about their experiences of completing the tools, reflecting on their problems and needs and their experiences of the conversations with a RN. In the focus groups, the RNs were asked about their experiences of the conversation tools and the conversations, as well as their perceptions of the patients’/family caregivers’ experiences. Participants were encouraged to speak freely, with follow-up questions prompting them to elaborate on their answers. The interview guides were developed by the research team, which has extensive experience of interviews and interview guides. All interviews were audio recorded and transcribed verbatim.

### 2.4. Data Analysis

Descriptive statistics were used to present the characteristics of the participants, as well as the number and percentage of completed conversation tools and conducted subsequent conversations. The data analysed were obtained from the documentation in patients’ medical records as well as from the RNs’ documentation of the conversations with family caregivers. In some cases, the tools were completed by patients or FCs and used as a basis for the conversations but failed to be saved for the study, but all conversations were registered.

Inductive as well as deductive content analysis of the manifest content was applied to the interviews [30,31]. Each interview was initially read repeatedly to gain a sense of what was being expressed. In the next step, phrases and paragraphs expressing content related to the aim were extracted and labelled inductively [30]. A codebook was developed in Excel for the documentation of the analysis. Due to the fact that the experiences were expressed for one tool at a time, the extracted data were documented per tool, which formed the qualitative results pertaining to acceptability. While labelling the data during the analysis, it emerged that the participants’ experiences of using the tools could be divided into the following categories: positive, negative, neutral and both positive and negative. We therefore continued with a deductive content analysis [31], which led to the data being categorised and quantified based on every participant’s experience of each tool. Two of the authors (A.M.K and L.V.E.) performed the main analysis and conducted reflexive memoing throughout the analysis process to increase dependability. The analysis was validated with two of the other authors (A.S. and J.W.) and later with all authors. All authors read and validated the entire result, with adjustments made until consensus was achieved.

## 3. Results

### 3.1. Participants

A total of 76 patients were assessed for eligibility. Of these, 11 did not meet the inclusion criteria, nine due to lack of Swedish language proficiency and two due to cognitive impairment, leaving 65 patients eligible for participation. Among the eligible participants there was a total attrition of 29 patients, due to declining participation (*n* = 17), administrative failures (*n* = 6), lack of response (*n* = 4) and cancelled or postponed transplantation (*n* = 2), resulting in 36 patients included in the study (55% participation rate). Three patients declined family caregiver participation, while all but one of the invited family caregivers agreed to participate, resulting in the inclusion of 32 family caregivers (97% participation rate). No eligibility criteria changes were made after commencement of recruitment. The characteristics of the 36 patients and 32 family caregivers are presented in Table 1. Among the participants, 16 patients and 15 family caregivers agreed to participate in an interview. The median duration of the interviews was 40 min (range 17–135 min) for patients and 18 min (range 9–24 min) for family caregivers. In total, 16 RNs participated in four focus group interviews: one at each of the two in-patient care units, one with “Champions” (selected RNs who had a central role in the project by functioning as a link between the centres and the research group) together with RNs from one of the out-patient care units and one with the HCT coordinators from both centres. The median duration of these interviews was 53 min (range 46–58 min).

### 3.2. Practicality of the Model

The analysis of the completed conversation tools and documentation of the subsequent conversations showed that most patients, family caregivers and RNs found the person-centred nursing model practical.

#### 3.2.1. Practicality of Using the ARNC Tool

The results indicate that the use of the ARNC tool and subsequent conversations were highly practical. Data show that among participating patients in in-patient care 86% (*n* = 31) completed the ARNC and 94% (*n* = 34) had a subsequent conversation with an RN. Furthermore, in out-patient care, 92% (*n* = 33) of participating patients completed the ARNC and 97% (*n* = 35) had a subsequent conversation with an RN.

#### 3.2.2. Practicality of Using the SNAP Tool

The use of the SNAP tool and subsequent conversations were moderately practical. Data show that 67% (*n* = 24) of the participating patients filled in the SNAP conversation tool and 78% (*n* = 28) had a subsequent conversation with an RN.

#### 3.2.3. Practicality of Using the CSNAT-I Tool

The use of CSNAT-I tool and subsequent conversations were highly practical. Data show that among participating family caregivers 94% (*n* = 30) completed the CSNAT-I conversation tool and 94% (*n* = 30) had a subsequent conversation with an RN.

### 3.3. Acceptability of the Nursing Model

#### 3.3.1. Overall Experiences of Using the Tools and Subsequent Conversations

The analysis of the interviews showed that most patients, family caregivers and RNs found the care model acceptable, expressing that using the tools and having the conversations was experienced as appropriate and satisfactory. In general, the conversation tools and conversations were perceived as separate parts of the care, not as parts of a complete model. In total, 78% of the participants were positive or both positive and negative about using the ARNC, 41% about the SNAP and 95% about the CSNAT-I (Table 2). However, some patients and family caregivers expressed neutral experiences, i.e., when no needs were identified the tools and conversations were perceived as less appropriate, while other patients and RNs described predominantly negative aspects of using the tools and the subsequent conversations. More detailed descriptions of participants’ experiences of using the three conversation tools and the conversations are presented below.

#### 3.3.2. Experiences of Using the ARNC Tool

Patients and RNs generally found using this tool appropriate and satisfactory. They experienced it as straightforward, simple and valuable, in admissions to both in-patient and out-patient care. Patients valued the relevance and understood the purpose of the domains, noting that the tool also enhanced communication with healthcare professionals and facilitated the monitoring of problems across various health domains. Patients also reported increased awareness of specific health issues and felt supported in managing symptoms. They valued the tool’s ability to encourage self-reflection on current and potential future needs, particularly regarding employment and financial matters as they relate to ongoing health issues. Information provided about additional support services, such as financial counselling, was particularly appreciated.

“I think all questions are relevant, I understand why the questions are being asked and feel they are appropriate for the situation.”

(Patient 2)

“Needs emerge that would not have been discussed otherwise, or that their biggest problem is not what you thought it would be.”

(RN, focus group 2)

“I have heard that they like it a lot, precisely because they are able to sit and reflect on the questions in the tools and become aware of what the real problem is.”

(RN, focus group 1)

The appropriateness of the tool experienced by both patients and RNs concerned the fact that the domains were perceived as relevant, facilitating an overview of which domains to focus on and identifying where patients needed more information and support. The tool enabled an open dialogue where patients felt more involved in choosing what to discuss and made it easier for them to put their feelings into words, which helped the RNs to understand patients’ needs and focus on important areas. Patients felt acknowledged and supported by the RN. RNs experienced that the conversations focused on the troublesome aspects, meaning that they did not have to spend much time on areas where the patient did not indicate any needs, which was timesaving, efficient and enabled them to adjust the care. The tool facilitated a good structure for their conversations with patients and allowed the RNs to offer self-care advice as well as support from other healthcare professionals. Patients also expressed satisfaction with the way in which the tool facilitated open conversations about sensitive domains such as sexuality and finances and made these conversations more approachable and comfortable, which some patients found empowering. RNs stated that using the tool was appropriate in terms of raising domains that RNs usually do not discuss. Some patients expressed ambivalence about the benefit of the tool because it made them think more about their health issues.

“It helps you realise which areas you need to focus on, a positive experience of filling in forms, not only important for healthcare but also important for yourself. It makes you think, become more aware of what you should keep an eye on, drinking, urinating, how your skin looks, moving around.”

(Patient 17)

“I think that many patients consider sexuality a problem, and that they have a lot of problems in that area, and it feels like an easier approach to that topic, which can perhaps be difficult to bring up, just like that.”

(RN, focus group 3)

“The biggest difference I’ve experienced is that you have a lot more conversations about family and relationships, and something that is completely new to me, finances. Something I have never talked to my patients about. Because for me it’s not a problem in inpatient care, but it obviously is.”

(RN, focus group 2)

RNs also reported that the tool was not experienced as appropriate in all situations, e.g., when patients were not interested in nor willing to share their feelings or experiences. RNs experienced that older patients and those with cognitive difficulties could find using the tool challenging, but that the participation of family caregivers was often valuable in such situations, as they could help describe the patient’s perspective. However, some RNs also observed that the presence of family caregivers during the conversations could restrict patients, especially in the domain pertaining to family and relatives. An unsatisfactory aspect experienced by RNs was when there was no possibility of being alone with the patient, which made it difficult to have a confidential conversation and follow up the identified needs.

“There are limits to what you are willing to talk about when you have another relative with you, so I felt that it was... a bit of a shame.”

(RN, focus group 1)

Some RNs experienced that the tool was not appropriate for patients who considered that they had no needs, as they found it pointless. Patients without major concerns also described a sense of superfluousness and some were unsure about the tool’s ability to highlight relevant issues in relation to the amount of information already provided during standard care. Some patients noted a lack of conversation after completion of the tool, which left them sceptical about the tool’s actual benefit. Some RNs experienced dissatisfaction when required interventions did not take place due to the RN’s lack of confidence or knowledge, as well as when there was a sense that a domain was inadequate.

“It was no help for me to become aware of troublesome problems, probably because I had no problems.”

(Patient 24)

“I know one patient who just said no, I don’t have any problems, so... Who thought it wasn’t worth filling in because there’s nothing bothering me.”

(RN, focus group 3)

#### 3.3.3. Experiences of Using the SNAP Tool

Patients experienced the SNAP tool as satisfactory with its comprehensive approach to addressing a broad range of concerns, which were often not initially considered by the patients themselves. The RNs’ mutual understanding was that the tool might be useful for this group of patients, as many of the domains were considered relevant.

Patients experienced that the domains within the tool were proof that the healthcare team was engaged beyond routine care. They found the conversations with RNs beneficial, as they helped to clarify and address domains raised in the tool. A segment of patients described their feelings about the appropriateness of the tool as neutral, acknowledging that while the tool was straightforward to use and included relevant domains, it did not alter their care process or reveal new insights. These patients acknowledged the potential appropriateness of the tool for others who might have more pressing or unaddressed needs, and for patients who were not active or lacked support. Some patients expressed that they had not experienced any impact or changes to their life situation or care from using the tool, which led to scepticism regarding its appropriateness and purpose, hence it was experienced as a bureaucratic exercise rather than a meaningful part of care. Concerns were also raised about the lack of follow-up on issues identified through the tool, leading to questions about its overall purpose and efficacy.

“It can definitely be a support. I didn’t need it myself, but it’s still good to know how it could have been, good to think for myself whether or not that was the case.”

(Patient 16)

“I think the basic idea is very good. I find it a little difficult to put it into a time perspective, when to use it. I think it’s a bit too early, as we’ve been using it right now, during discharge conversations.”

(RN, focus group 3)

A few patients expressed a need for the tool to be more adaptable to their changing health circumstances, suggesting that its content seemed too general or disconnected from their current situation. Patients who already felt well-supported through existing healthcare processes or personal support systems suggested that the tool might be more effective if introduced at varying stages of care, as many participants noted that their needs were already being met through standard care interactions. The patients pointed out that its appropriateness seemed greater for post-discharge scenarios rather than during an active hospital stay, which would potentially lead to more thoughtfulness and reflective conversations after adjustment to their home environment. RNs also expressed that the timing of using the tool was not optimal and believed it was used too early in the care process. Suggestions about suitable time points for the tool were raised and the RNs thought it would be relevant to use 2–3 months, or even 6–12 months after transplantation. Furthermore, the RNs stated that they had not really understood how to use the tool and how to address some of the domains. Additionally, in some cases the tool was used at the same time as the ARNC, which the RNs found pointless.

“It is vague, unclear, I cannot give a precise answer, I do not really understand the purpose of the form, what the aim is, I mostly guess when filling it in. Difficult when leaving hospital, better after six months, a year.”

(Patient 2)

“The SNAP tool is perhaps the most difficult to get right. I have tried a few in inpatient care, and I think it ends up completely wrong there. The patients themselves... everyone I have spoken to has said, this is too early, I would like to do this when I get home and have been at home. In outpatient care, I think it should be done a little later, when the patient has been home for a while.”

(RN, focus group 1)

#### 3.3.4. Experiences of Using the CSNAT-I Tool

Family caregivers and RNs generally found the tool appropriate and satisfactory. Several family caregivers experienced that the tool itself was user-friendly and identified domains in which they needed more support, such as ‘Dealing with your feelings and worries’ and ‘Knowing who to contact if you are concerned’, highlighting the fact that these areas might not have otherwise been raised. Several family caregivers experienced that the tool helped them to voice their emotions and concerns more openly, reducing anxieties about the ill person’s condition. The conversation validated their experiences and emotional burden, providing them with comfort and a sense of being included in the patient’s care. The use of the tool offered an opportunity to feel acknowledged and supported by the RNs. One common benefit experienced by family caregivers was that the systematic use of tools with conversations improved communication between family caregivers and healthcare professionals and they appreciated receiving practical advice and guidance on available resources to address their needs. Some family caregivers, however, found that the tool and the conversation were supplementary rather than essential because they did not align with their needs or were unnecessary due to the fact that they already had a functioning support system. A few family caregivers expressed that some domains were not relevant, e.g., ‘Practical help in the home’ and ‘Providing personal care for your relative’, especially if they were not living with the patient or providing hands-on care. Some highlighted that using the tool raised concerns they had not previously considered, which led to increased anxiety. However, they acknowledged that this might be useful for other family caregivers.

“It was good to be able to put things into words, to talk about what you are thinking about, and the tool felt relevant; I’m sure everyone needs that. I think it’s good that I am involved as a relative and that I have the opportunity for conversation and support.”

(Family caregiver 11)

“Yes, it was really great. I think it feels very reassuring to be able to participate in something like this, because then you feel that you are involved in a different way. Yes, well, it’s just that you feel included in some way, perhaps you gain a greater understanding of it because you think a little more about these issues and so on.”

(Family caregiver 15)

All RNs agreed that it was satisfying to have conversations with family caregivers based on the CSNAT-I tool. Most RNs stated that the tool provided a good structure for the conversation and found it appropriate to ask family caregivers about their support needs. The vast majority experienced that they had sufficient knowledge to be able to give advice and manage what arose in the conversations. However, at the same time they revealed that the most common domain that family caregivers wanted to discuss was worries about what to expect in the future, which felt uncomfortable as it is impossible to foresee the future; thus, the RNs were unable to provide an answer to support the family caregiver. Several RNs stated that giving the family caregivers a previously developed brochure about available support was helpful in the conversation and family caregivers appreciated this information about the availability of extra resources. One RN expressed that although all domains in the tool were not relevant on every occasion, it provided an opportunity to inform the family caregivers about where they could seek help at a later stage of the transplantation process. However, RNs revealed that these conversations could be tough and challenging due to the emotional impact on family caregivers. A few RNs also stated that it was difficult to ensure that the conversation did not take too long, because some family caregivers had a great need of support. All RNs who conducted these conversations were HCT coordinators and they expressed that someone else might be better suited to conducting these conversations due to their lack of time and a perception that this was not really part of their work. The HCT coordinators also experienced confidentiality issues in that it could feel as if they were keeping secrets from the patient after the conversation with the family caregiver.

“Yes, but using this tool provides a good structure. I think it also makes the family caregiver think a little more. That someone puts into words certain things that you might not have thought of otherwise.”

(RN, focus group 4)

“It was clear that the family caregivers appreciated having the conversations and feeling that they were seen. Family caregivers were very grateful for having had the conversation and made comments like, ‘yes, but now I have a better understanding of it. Now I feel better.’”

(RN, focus group 4)

“It’s about the future, what to expect. That was probably the most... Actually... I think that’s what concerns me the most.”

(Family caregiver 12)

The use of the CSNAT-I to engage family caregivers in the care process before patients’ admission to inpatient care was generally well received. Some family caregivers and RNs felt that the conversations occurred too early, addressing domains that were not immediately relevant, and with a need for follow-up of CSNAT-I conversations.

“In the best of worlds, two calls would have been best. I wonder if it wouldn’t be better to have a second one afterwards. I get the feeling that they might need more support then.”

(RN, focus group 4)

## 4. Discussion

The results show that our person-centred nursing model targeting the needs of patients and family caregivers in the context of allo-HCT is feasible in terms of practicality and acceptability from the perspectives of patients, family caregivers and RNs. Using conversation tools and subsequent conversations facilitates the creation of a structure for the assessment of patient and family caregiver needs, where the individual situation of each patient and family caregiver is seen holistically. The model was largely conducted as intended and the two conversation tools, the ARNC and CSNAT-I, were used as intended and generally perceived as positive. However, the SNAP was less used as intended and perceived less positive in this context. In general, the conversation tools and conversations were perceived as separate parts of the care, not as parts of a complete model. In addition, challenges emerged regarding the issue of who is best suited to conduct conversations with family caregivers.

The structured nature of our nursing model facilitated discussions that allowed patients and family caregivers to express their concerns and needs, which is consistent with previous research demonstrating the value of person-centred frameworks in enhancing communication and engagement in cancer care [32]. Structured tools such as the ARNC and SNAP have been validated in previous studies for their effectiveness in identifying patient needs [22,23]. Our findings confirm their utility in facilitating meaningful conversations, particularly in addressing broad issues.

The positive experience of the ARNC by both patients and RNs in the present study supports the comprehensive goal of person-centred nursing to focus the care on patients’ unique needs and preferences [20,21]. Our findings suggest that the sensitive domains included in the ARNC, such as sexuality, appearance and financial issues, may require additional attention in clinical practice. Although these domains were highlighted by the tool, RNs were feeling unprepared or uncomfortable discussing them, despite their central role in the psychological and social needs described in previous research [3,9]. Targeted training, practical resources and clinical guidelines have been shown to be essential for bridging this gap and can significantly enhance healthcare professionals’ confidence and competence in addressing sensitive topics [33,34]. Our nursing model includes a web-based education for healthcare staff about person-centred nursing as well as the nursing model itself with its three tools. However, the results of this feasibility study indicate a need for additional or more detailed education and training about sensitive topics.

The SNAP tool was perceived as less useful during the timeframe in which it was used in this study, with both patients and RNs suggesting it might be more effective if introduced later in the care trajectory, such as during the rehabilitation phase. This finding aligns with previous research showing that patients’ needs evolve over time and that careful consideration of when to introduce supportive tools is crucial [23]. Adjusting the timing of such interventions could enhance their relevance and utility in addressing the evolving challenges faced by patients undergoing allo-HCT. The ARNC and the SNAP were used in close connection after in-patient care, with the ARNC about patients’ problems and the SNAP about patients’ support needs. One assumption is that patients are not used to being asked about their needs and that they already receive sufficient attention from healthcare professionals, therefore the SNAP tool was experienced as superfluous by both patients and RNs.

The findings show positive experiences from both family caregivers and RNs that the CSNAT-I provided family caregivers with an opportunity to articulate their concerns and facilitated collaboration between family caregivers and healthcare professionals, as described in our previous feasibility study of the CSNAT-I in this context [28]. This also aligns with the three core components of our person-centred nursing model, narratives, partnerships and shared care plans [21]. Our nursing model recognised and validated family caregivers’ experiences, helping them feel acknowledged and emotionally prepared, which in turn strengthened their ability to provide effective support to the patient. Some family caregivers did not live together with the patient and therefore found certain domains regarding physical care for the patient less relevant. Most family caregivers in the present study were positive (63% positive and 31% positive and negative) about having a CSNAT-I conversation with a RN, while all family caregivers (100%) in our first feasibility study were satisfied about using the CSNAT-I [28]. This difference is likely due to the fact that in the present study family caregivers had only one conversation before the transplantation, compared to one before and one about 6 weeks after transplantation in the earlier study [28]. Two conversations over time seem to better provide family caregivers with support and increase their well-being. Improved well-being of family caregivers is beneficial not only for the individual family caregiver but also reduces family caregivers’ healthcare utilization and costs, benefiting society [35]. The negative experiences regarding using the CSNAT-I conversations in the present study were mainly from the RNs conducting these conversations. The RNs experienced the conversations as emotionally demanding, time-consuming and outside the scope of these HCT coordinators’ work assignment. Involving other professionals, such as social workers or psychologists, could help distribute responsibilities while maintaining the model’s holistic approach [18,19] and enhance care quality but also support healthcare providers, potentially reducing burnout and improving their sense of efficacy [36]. The CSNAT-I has demonstrated its capacity to identify diverse needs, ensuring that all family caregivers are offered support, regardless of their circumstances and therefore universal implementation of the tool could improve equity in access to family caregiver support.

A key finding was that participants expressed a lack of follow-up on needs identified through the tools and conversations. This highlights a critical gap in integrating the tools into a broader system of education, care planning and follow-up, leading to the risk of identified needs being overlooked and failure to fully achieve the holistic goals of the nursing model. Using the SNAP and CSNAT-I requires training, which the participating RNs received, but apparently it was insufficient, showing the importance of repeated and continuous training. Further, it is imperative to engage RNs in the clinic in further collaboration to develop a sustainable framework that delineates responsibility for follow-up, as well as the methods, timing, and documentation of these actions. Research has consistently shown that structured follow-up documentation significantly improves continuity of care and patient satisfaction [37]. Developing clear documentation that assigns accountability for revisiting identified needs could help to ensure that patient and family caregiver concerns are effectively integrated into ongoing care. Clearer forms of follow-up documentation are necessary to improve continuity of care, enhance patient and family caregiver satisfaction and ensure that identified needs, including emotional ones, are systematically addressed in subsequent care interactions.

### 4.1. Strengths and Limitations

This study has several strengths that contribute to its robustness and relevance. Based on a sample from two allo-HCT centres, it captures insights from geographically diverse settings, which strengthens the transferability of the findings. One strength of the study is that inclusion was made consecutively with few exceptions, to ensure equity in access to support. The use of validated tools (ARNC, SNAP and CSNAT-I) enabled a structured and systematic approach to identifying patient and family caregiver needs, while the quantitative and qualitative design allowed for a comprehensive evaluation of the model’s feasibility. Furthermore, the involvement of champions in the planning and development of the model enhanced its clinical relevance and practicality, aligning with participatory research principles that emphasize the value of engaging frontline professionals in intervention design.

Despite these strengths, the study also has significant limitations. Certain patient groups were excluded, such as those with language barriers and cognitive impairments, and not all wanted to participate, which may reduce the transferability and generalizability of the findings. One limitation is that characteristics of the participating RNs, such as age and work experience, were not collected and can therefore not be presented. Another limitation of the study is the 55% participation rate, which may lead to uncertainty about what conclusions can be drawn. Despite the relatively low number of participants, we believe that our feasibility study shows interesting and important results that can be used in both clinical practice and further studies. In order to achieve a higher number of participants, the information and inclusion procedures should be reviewed and revised. The fact that the interview guides were not pilot tested, and that no inter-coder reliability statistic was calculated can be seen as a limitation. However, as the interview guide as well as the results from the content analysis were discussed extensively within the research group and revised until consensus was reached, this can be considered valid. Another limitation was that it was not possible in clinical practice to perform two conversations with family caregivers, one before and one 6 weeks after the allo-HCT, which was done and positively evaluated in our previous feasibility study with family caregivers [28].

### 4.2. Recommendations for Further Research

To build on the findings of this study, future research should focus on measuring the model’s effectiveness for assessing and addressing patients’ and family caregivers’ problems and needs and if the model leads to shared care planning, in the present sample as well as later in a multi-centre study. Longitudinal studies are particularly needed to assess the long-term impact of the model on patient and family caregiver outcomes, especially during the rehabilitation phase when needs evolve significantly. Additionally, expanding the study to include varied demographic and other clinical contexts would strengthen the evidence for its effectiveness and generalizability.

Studies investigating the economic impact of family caregiver involvement in patient care could offer valuable insights into the cost-effectiveness of supporting family caregivers in allo-HCT. Research in this area would provide a stronger foundation for advocating for family caregiver-focused interventions, aligning with the broader goals of improving healthcare efficiency and reducing societal costs.

### 4.3. Implications for Clinical Practice

The results indicate that the use of conversation tools, especially the ARNC and the CSNAT-I aids RNs to assess patients’ and family caregivers’ problems and needs, while considering each person’s situation holistically. However, there are practical challenges involved in using our nursing model. To adequately address the holistic needs identified in the conversations, RNs require further targeted training, access to practical resources and clear clinical guidelines on when to use each tool and avoid simultaneous use, to cope with the holistic needs identified in the conversations and be part of a multidisciplinary team that can address them. Additionally, their role within the multidisciplinary care team must be supported and clarified, and the support to family caregivers shared with psychosocial professionals. In addition, there is a need to enhance the follow-up of assessed and addressed needs by improving continuity of care and developing clearer care plans in a partnership between the RN, the care team, the patient and family caregiver. By addressing these practical challenges, our nursing model has the potential to serve as a sustainable and effective approach to person-centred nursing in allo-HCT, improving outcomes for both patients and family caregivers and reduce preventable suffering, while supporting healthcare providers in delivering holistic care.

## 5. Conclusions

This feasibility study highlights the fact that our nursing model is promising for delivering person-centred nursing to patients and family caregivers during allo-HCT. The results demonstrate the high practicality and acceptability of many parts of the model. This way of working offers a structured approach to addressing the complex needs of this group of patients and their family caregivers. However, challenges were identified that require further attention, including the fact that one tool (the SNAP) was found to be less practical and acceptable when tested in this study; furthermore, it was found that RNs need training, clear clinical guidelines for addressing sensitive topics and stronger systems for follow-up. Additionally, resource allocation is needed to include family caregivers in our model. With further development based on the results of this feasibility study, our person-centred nursing model has the potential to enhance the quality of care and support provided to patients and their families. To conclude, the model offers a promising foundation for person-centred nursing in the allo-HCT context, but for the model to be sustainable, investment in education, multidisciplinary integration and incorporation of the model into standard care practices are needed.

## Figures and Tables

**Table 1:** Characteristics of the participating patients and family caregivers.

	Patients *n* = 36	Family Caregivers *n* = 32
**Sex, *n* (%)**		
Women	14 (39)	24 (75)
Men	22 (61)	8 (25)
**Age at allo-HCT**		
Median (min-max)	55 (19–77)	52 (21–72)
**The family caregivers’ relationship to the patient**		
Partner		22 (69)
Parent		3 (9)
Child		3 (9)
Sibling		4 (13)
**Education, *n* (%)**		
Lower education	13 (42)	15 (47)
Higher education (College/University)	18 (58)	17 (53)
**Living situation, *n* (%)**		
Living with someone	22 (71)	27 (84)
Living alone	7 (23)	5 (16)
Missing	2 (6)	-
**Children under 18, *n* (%)**		
Yes	9 (29)	9 (28)
No	21 (68)	22 (69)
Missing	1 (3)	1 (3)
**Country of birth, *n* (%)**		
Sweden	26 (84)	29 (91)
Other	5 (16)	3 (9)

Allo-HCT = Allogeneic Haematopoietic Stem Cell Transplantation.

**Table 2:** The number of patients, family caregivers and RNs who experienced using the different tools in a conversation as positive, neutral, negative or both positive and negative as revealed by the interviews.

		Positive *n* (%)	Neutral *n* (%)	Negative *n* (%)	Positive & Negative *n* (%)
**ARNC**	Patients, *n* = 16	7 (44)	2 (12.5)	5 (31)	2 (12.5)
	RNs, *n* = 16	10 (62.5)	0 (0)	0 (0)	6 (37.5)
**SNAP**	Patients, *n* = 16	2 (12.5)	5 (31)	5 (31)	4 (2.5)
	RNs, *n* = 6	0 (0)	0 (0)	3 (50)	3 (50)
**CSNAT-I**	Family caregivers, *n* = 16	10 (63)	1 (6)	0 (0)	5 (31)
	RNs, *n* = 5	0 (0)	0 (0)	0 (0)	5 (100)

RN = Registered Nurse.

## Data Availability

The data presented in this study are not publicly available due to ethical and legal considerations related to participant confidentiality. However, de-identified datasets may be made available from the corresponding author and principal investigator upon reasonable request.

## Abbreviations

The following abbreviations are used in this manuscript:

Allo-HCT	Allogeneic Haematopoietic Stem Cell Transplantation
ARNC	The Assessment of Rehabilitation Needs Checklist
EBCD	Experience-based Co-Design
CSNAT-I	The Carer Support Needs Assessment Tool Intervention
GPCC	Gothenburg Centre for Person-centred Care
IPC	In-patient care
MRC	Medical Research Council
OPC	Out-patient care
RN	Registered Nurse
SNAP	The Support Needs Approach for patients
